# An Antimicrobial Peptide-Loaded Gelatin/Chitosan Nanofibrous Membrane Fabricated by Sequential Layer-by-Layer Electrospinning and Electrospraying Techniques

**DOI:** 10.3390/nano8050327

**Published:** 2018-05-14

**Authors:** Yuzhu He, Yahui Jin, Xiumei Wang, Shenglian Yao, Yuanyuan Li, Qiong Wu, Guowu Ma, Fuzhai Cui, Huiying Liu

**Affiliations:** 1 Department of Oral and Maxillofacial Surgery, School of Stomatology, Dalian Medical University, Dalian 116044, China; hyz138_2645@163.com (Y.H.); jyh9012@126.com (Y.J.); yuanyuan0154@163.com (Y.L.); Mgw64024@163.com (G.M.); 2 State Key Laboratory of New Ceramics and Fine Processing, School of Materials Science and Engineering, Tsinghua University, Beijing 100084, China; wxm@mail.tsinghua.edu.cn (X.W.); shenglian_yao@ustb.edu.cn (S.Y.); cuifz@mail.tsinghua.edu.cn (F.C.); 3 Zhejiang Provincial Hospital of Chinese Medicine, Hangzhou 310018, China; 4 School of Life Sciences, Tsinghua University, Beijing 100084, China; wuqiong@mail.tsinghua.edu.cn

**Keywords:** GBR membrane, antimicrobial peptides, electrospinning, electrospraying, PLGA microsphere

## Abstract

Guided bone regeneration (GBR) technique is widely used in the treatment of bone defects caused by peri-implantitis, periodontal disease, etc. However, the GBR membranes commonly used in clinical treatments currently have no antibacterial activity. Therefore, in this study, sequential layer-by-layer electrospinning and electrospraying techniques were utilized to prepare a gelatin (Gln) and chitosan (CS) composite GBR membrane containing hydroxyapatite nanoparticles (nHAp) and antimicrobial peptide (Pac-525)-loaded PLGA microspheres (AMP@PLGA-MS), which was supposed to have osteogenic and antibacterial activities. The scanning electron microscope (SEM) observation showed that the morphology of the nanofibers and microspheres could be successfully produced. The diameters of the electrospun fibers with and without nHAp were 359 ± 174 nm and 409 ± 197 nm, respectively, and the mechanical properties of the membrane were measured according to the tensile stress-strain curve. Both the involvement of nHAp and the chemical crosslinking were able to enhance their tensile strength. In vitro cell culture of rat bone marrow mesenchymal stem cells (rBMSCs) indicated that the Gln/CS composite membrane had an ideal biocompatibility with good cell adhesion, spreading, and proliferation. In addition, the Gln/CS membrane containing nHAp could promote osteogenic differentiation of rBMSCs. Furthermore, according to the in vitro drug release assay and antibacterial experiments, the composite GBR membrane containing AMP@PLGA-MS exhibited a long-term sustained release of Pac-525, which had bactericidal activity within one week and antibacterial activity for up to one month against two kinds of bacteria, *S. aureus* and *E. coli*. Our results suggest that the antimicrobial peptide-loaded Gln/CS composite membrane (AMP@PLGA-MS@Gln/CS/nHAp) has a great promise in bone generation-related applications for the unique functions of guiding bone regeneration and inhibiting bacterial infection as well.

## 1. Introduction

Guided tissue regeneration (GTR) generally refers to a surgical procedure, assisting tissue regeneration by utilizing a physical barrier membrane to maintain sufficient space and prevent surrounding undesired fibrous tissue intrusion [1]. Guided bone regeneration (GBR) was proposed based on GTR to specifically support bone regeneration in diverse applications (e.g., treatment of periodontal disease, alveolar bone augmentation, cranial bone regeneration, as well as repairing several other types of bone defects) [2]. An ideal GTR/GBR membrane is not only a physical barrier, but also delivers bioactive stimuli to guide tissue regeneration. Therefore, the development of multifunctional GTR/GBR membranes is essential for fulfilling the requirements in various clinical applications [3].

Based on whether it is degradable or not, the current widely used membranes can be categorized into two types: non-absorbable and absorbable membranes [4,5]. A non-absorbable membrane is usually made of materials like expanded polytetrafluoroethylene (e-PTFE) and pre-formed titanium mesh. Meanwhile, the materials used for absorbable membranes, such as polylactic acid (PLA), polyglycolic acid (PGA), PLGA copolymer, and collagen, have good biodegradability and biocompatibility, obviating the necessity of a second operation to remove them [6,7,8]. At present, these membranes, which are extensively utilized in the clinic, are able to meet the requirements for biocompatibility, mechanical barriers, and permeability, as well as for clinical maneuverability. However, they are not satisfactory, because of their limited osteogenic capacity and antimicrobial activity.

It is well-known that bacterial infection is a common complication associated with injury, and may aggravate the injury and affect tissue repair. Besides, bacterial adhesion to biomaterials has been one of the main reasons of implants failure. For instance, peri-implantitis, a type of destructive inflammatory process affecting the soft and hard tissues surrounding dental implants, is associated with the formation of a bacterial biofilm and compromises immunity at the implant/tissue interface that may extend to progressive loss of alveolar bone around the implant and dental implant failure. Therefore, developing a novel multifunctional membrane with combination of osteogenic and antibacterial activities is highly demanded for the infection-associated bone repair.

Antimicrobial peptides (AMPs) that exist widely in animals and plants have been demonstrated with a broad spectrum of activity against bacteria, viruses, and fungi. Unlike conventional antibiotic drugs that have the concern of antibiotic resistance, AMPs exhibit little drug resistance, as well as other great advantages, involving high efficiency, rapid sterilization, small molecular weight, appropriate thermal stability, no immunogenicity, and low sensitivity to enzymatic hydrolysis, and have therefore been widely studied as an alternative to traditional antibiotics in recent years. Their antibacterial mechanism involves the antibacterial peptides with net positive charges attracting the negative electrostatic charges on the surface of bacteria and adhering to the bacterial cell membrane, inserting themselves into it, which leads to cell membrane rupture, destruction of the integrity of the bacterial cell membrane, and ultimately cell death [9,10]. To date, hundreds of antimicrobial peptides have been discovered, which are generally cationic peptides with 10–50 amino acids in length. Recently, a new short Tryptophan (Try)-rich peptide (Ac-KWRRWVRWI-NH2), designated Pac-525, has exhibited a strong ability to insert into membranes with improved activities against both bacteria and fungi, including Streptococcus sanguis (*S. sanguis*), Fusobacterium nucleatum (*F. nucleatum*), and Porphyromonas gingivalis (*P. gingivalis*) [11,12,13]. Try has a strong ability to insert into the bacterial membranes and help Try-rich peptides go into membranes to affects lipid polymorphism [14,15]. The Pac-525 could bind to negatively charged phospholipid vesicles strongly and destabilize the microbial membrane. In addition, it’s been reported that Pac-525 exhibits good resistance to both Gram-positive and Gram-negative bacteria. The lipopolysaccharides outside the Gram-negative bacteria and acidic polysaccharides outside the Gram-positive bacteria confer a net negative charge to the surfaces of the bacteria [12,16]. Additionally, it’s been proven that Pac-525 has high biosafety and low cytotoxicity [12]. In addition, it contains inhibitory influence on most of the pathogenic bacteria; however, it has no significant inhibitory effect on the oral probiotics, which is beneficial for the maintenance of oral micro ecological balance. Therefore, Pac-525 was applied in the proposed GBR membrane to improve its antibacterial activity. However, AMPs are unstable and easily degraded in vivo. In order to ensure the long-term activity of AMPs, poly lactic-co-glycolic acid (PLGA) microspheres were used for protection and sustained release [17]. Meanwhile, the cationic chitosan also possesses appropriate antibacterial effects, and simultaneously neutralizes the acidic degradation products of PLGA.

In this study, an antimicrobial peptide-loaded multifunctional GBR membrane is designed and prepared by sequential layer-by-layer electrospinning and electrospraying techniques. The nanofibrous membrane that was fabricated by electrospinning a composite of gelatin (Gln) and chitosan (CS) with or without the hydroxyapatite nanoparticles (nHAp) comprised two layers, barrier layer and osteogenic layer. The AMP-loaded PLGA microspheres (AMP@PLGA-MS) were uniformly dispersed throughout the whole membrane through alternant electrospraying to enable the membrane a sustained antibacterial activity. The electrospinning and electrospraying techniques were firstly combined together to make a layer-by-layer microspheres-embedded nanofiberous membrane. The rat bone marrow mesenchymal stem cells (rBMSCs) are cultured on the membrane to estimate in vitro biocompatibility as well as osteogenic activity. Moreover, the drug release profile is examined for up to one month. Besides, the antibacterial activity of the membrane against Staphylococcus aureus (*S. aureus*) and Escherichia coli (*E. coli*) were assessed as well.

## 2. Materials and Methods

### 2.1. Materials

Antimicrobial peptide Pac-525 (Ac-KWRRWVRWI-NH2), with purity of more than 99%, was custom-synthesized by Qiangyao Bio-Technology Co., Ltd. (Shanghai, China). In addition, PLGA (75:25, molecular weight (*M*_w_) = 5 × 10^4^) was purchased from Medical Equipment Research Institute (Jinan, China). Chitosan (*M*_w_ = 1 × 10^5^~3 × 10^5^) was purchased from the Bailingwei Science and Technology Co., Ltd. (Beijing, China). Gelatin of bovine origin was purchased from Sigma-Aldrich (Burlington, VT, USA). The nHA was purchased from Beijing Allgens Medical Science Technology Co., Ltd., (Beijing, China). The rBMSCs were obtained from Cyagen Biosciences Co., Ltd. (Guangzhou, China). Dulbecco’s Modified Eagle’s medium (DMEM), fetal bovine serum (FBS), antibiotics, and trypsin-EDTA were purchased from GIBCO Invitrogen Corporation/Life Technologies Life Sciences (Carlsbad, CA, USA). *S. aureus* (ATCC 6538) and *E. coli* (ATCC 25922) were obtained from Peking University Hospital of Stomatology and used as an example of Gram-positive and Gram-negative bacteria, respectively. All other chemicals used were of analytical grade and were obtained from Chemical Reagent Co., Ltd. (Beijing, China).

### 2.2. Methods

#### 2.2.1. Fabrication of the Nanofibrous Membranes

**Figure 1:**
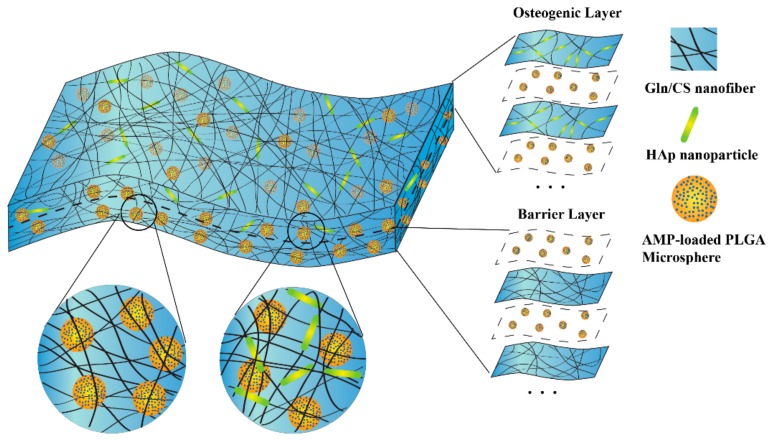
Schematic diagram of the fabrication process and structure of the AMP@PLGA-MS@Gln/CS/nHAp composite membrane by sequential layer-by-layer electrospinning and electrospraying. This biodegradable membrane consists of two layers: the barrier layer (Gln/CS nanofibers) and osteogenic layer (Gln/CS/nHAp nanofibers). The AMP-loaded PLGA microspheres were electrosprayed alternately during the electrospinning, and were therefore embedded within the membrane.

Gln/CS solution was prepared by dissolving Gln and CS in acetic acid (1:4) to form a homogeneous solution with a final concentration of 140 mg/mL by magnetic stirring overnight, which was abbreviated as S1. Meanwhile, nHAp powder was added to S1 solution and sonicated on ice using an ultrasonic crasher (Scientz-IID, Ningbo Science Biotechnology Co., Ltd., Ningbo, China) at a power of 300 W for 1 min to form a uniform suspension, which was abbreviated as S2. The PLGA solution was prepared by adding into chloroform at a concentration of 60 mg/mL with continuous stirring for at least 2 h. Additionally, 2.5 mg Pac-525 was dissolved in 50 μL of deionized water and then added to 1 mL of the PLGA solution to form a W/O emulsion by sonication on ice using an ultrasonic crasher at a power of 300 W for 20 s, which were abbreviated as P. The solutions of S1, S2, and P were then electrospun or electrosprayed layer by layer (Figure 1). The electrospinning and electrospraying were conducted alternately on the same collection plate by exchanging the syringes and adjusting the parameters. In brief, the S1 or S2 solution was poured into a 5 mL syringe and then erupted through a blunt stainless steel nozzle (inner diameter = 0.34 mm) to form a stable stream flow under an electrostatic force at a constant flow rate of 0.4 mL/h using an injection pump. Aluminum foil paper was used as a collection plate. The applied voltage to the nozzle tip was 20 kV, and the distance between the nozzle tip and the aluminum collection plate was 15 cm [18,19]. During electrospinning, the solution P was alternatively electrosprayed, forming PLGA microspheres embedded inside the interwoven Gln/CS nanofibrous network. The flow rate of solution P was 1 mL/h, the applied voltage to the nozzle tip was 5.5 kV, and the distance between the nozzle tip and the aluminum collection plate was 20 cm, as well. All these processes were carried out under ambient atmosphere [20]. According to the sequences of electrospinning and electrospraying, the barrier and osteogenic layers were denoted as (S1/P)n and (S2/P)n, respectively. The organic solvent was removed during the electrospinning process by evaporation, leaving membranes and solid microspheres on the collection plate. The membranes were cross-linked by immersing in the EDC/NHS (100 mM:25 mM) solution in 90% alcohol at −4 °C for 6 h, washed with deionized water for 3 times, and then freeze-dried for 24 h to remove the residual solvent.

#### 2.2.2. Characterization of the Nanofibrous Membranes

The surface morphology of the electrospun nanofibrous membrane was studied under a field emission scanning electron microscope (FE-SEM, Carl Zeiss, Oberkochen, Germany). In brief, the samples were washed twice using phosphate-buffered saline (PBS), and then fixed in 4% glutaraldehyde overnight. After that, the samples were again washed with PBS, dehydrated with graded concentrations of ethanol (30%, 50%, 70%, 80%, 90%, 95%, and 100%), exchanged twice in tertiary butyl alcohol/ethanol (1:1) and tertiary butyl alcohol for 10 min each, and then freeze-dried. Finally, the membranes were sputter-coated with gold (JEOL JFC-1200 Fine Coater, Tokyo, Japan) and observed under SEM at an accelerating voltage of 10 kV. Diameters of the electrospun fibers were analyzed by e-ruler for measuring SEM images. Hydrophilicity of the membrane was undertaken by water contact angle (WCA) measurement using VCA Optima Surface Analysis system (AST products Inc., Billerica, MA, USA). The tensile mechanical property of the membrane samples with the size of 25 mm × 5 mm was tested by using a universal testing machine (Sintech Renew 1121, Instron Engineering Corp., Canton, MA, USA) with a 10-N load cell. The crosshead speed was set at 10 mm/min. Tensile strength was calculated based on the obtained stress-strain curve [21]. The test was repeated 6 times for statistical analysis.

#### 2.2.3. Cell Culture

The coverslips with electrospun nanofibrous Gln/CS were sterilized in 75% ethyl alcohol under ultraviolet (UV) irradiation for 24 h for the following cell culture. The rBMSCs (passage 6–8) were cultured in DMEM supplemented with 10% (*v*/*v*) FBS, 100 IU/mL penicillin, and 100 IU/mL streptomycin in a humidified incubator at 37 °C and 5% CO_2_.

#### 2.2.4. Cell Proliferation Assay

The rBMSCs were seeded on the electrospun membranes and coverslips (as control) in a 24-well cell culture plate at a density of 1.2 × 10^4^ cells/well for up to one week. The cell numbers after 1, 4, and 7 days of culture were measured using Cell Counting Kit-8 (CCK-8) (Beyotime Institute of Biotechnology, Jiangsu, China). Briefly, after discarding the culture medium and washing with PBS, 100 μL of CCK-8 solution was added to each well cell culture plate and incubated for 3 h at 37 °C. The incubated solution was then transferred to a 96-well cell culture plate and measured using a microplate reader at 450 nm.

#### 2.2.5. Alkaline Phosphatase (ALPase) Activity Assay

As an early marker of osteogenic differentiation, the ALPase activities of the cells were examined using an ALP assay kit (Beyotime Institute of Biotechnology, Nanjing, China) following the manufacturer’s instructions. The cells were cultured in the osteogenic culture medium containing DMEM, 10% FBS, 1% PS, 10 mM β-Glycerophosphate disodium salt hydrate, 50 μM ascorbic acid, and 0.1 μM dexamethasone for up to 14 days. At the indicated time point, the samples were washed with PBS (pH 7.4), and then lysed in 1 × RIPA buffer (50 mM Tris-HCl, 150 mM NaCl, 0.25% deoxycholic acid, 1% NP-40, and 1 mM EDTA) using a protease inhibitor cocktail tablet (Roche, Mannheim, Germany) for 20 min on ice. Each lysate was centrifuged at 4 °C for 10 min to remove the cell debris. The supernatant was then incubated with p-nitrophenyl phosphate (PNPP). The optical density was measured by using a microplate reader at 405 nm. The ALPase activity was expressed in terms of units per gram of protein.

#### 2.2.6. Cell Morphological Examination

The morphological examination of in vitro cultured rBMSCs on all the membranes were performed after 1, 4, and 7 days of cell culture using SEM and confocal laser scanning microscopy (CLSM). The samples for SEM were prepared as previously indicated in Section 2.2.2. For CLSM (IX-71, Olympus Co., Ltd., Shinjuku, Tokyo, Japan) exanimation, the cells were fixed with 4% paraformaldehyde for 15 min, permeabilized with 0.1% Triton X-100 for 5 min at room temperature, and then stained with 4′,6-diamidine-2′-phenylindole dihydrochloride (DAPI) (Sigma-Aldrich Co., LLC., Rockville, MD, USA) for nucleus and Alexa Fluor 546 phalloidin (Invitrogen, CA, USA) for F-actin.

#### 2.2.7. In Vitro Release Profile of Antimicrobial Peptide

In order to investigate the release profile of Pac-525 from the AMP@PLGA-MS@Gln/CS/nHAp bilayer membrane, the membranes (10 mm × 10 mm) were immersed in 1 mL PBS (pH = 7.4), and incubated in a shaking bath (Model THZ-C, Taicang Laboratorial Equipment Factory, Suzhou, China) at 60 rpm and 37 °C. At each predetermined interval, 0.5 mL of supernatant buffer was collected and stored at −80 °C. And equal volume of fresh PBS was supplemented to keep the same total volume. The drug concentration was measured by a microplate reader at 280 nm. Mean and standard deviation (SD) of the results from three independent experiments were calculated for statistical analysis.

#### 2.2.8. Antibacterial Experiment

The antibacterial activity of the membranes against *S. aureus* and *E. coli* was evaluated by inhibition zone and inhibition ratio assays. *S. aureus* and *E. coli* were cultured in a Luria-Bertani (LB) medium containing tryptone (10 g/L), yeast extract (5 g/L), and NaCl (10 g/L) at 37 °C overnight. The inhibition zone assay was performed using the agar diffusion method (Oxford cup method). Briefly, bacteria were uniformly distributed on the surface of the agar plate, and then Oxford cups with inside diameter of 6 mm were placed onto the middle of the agar plates. After incubating at 37 °C for half an hour, 0.1 mL of each collected supernatant was added into the Oxford cup. PBS served as a negative control, and 1 mg/mL of Pac-525 solution served as a positive control. The inhibition zones that formed around the cylinders were measured after 16–18 h of incubation.

To evaluate the inhibition ratio, 800 μL of bacteria solution (10^5^ CFU/mL) was mixed with 200 mL of testing solution (supernatants were collected after one-week or four-week release for the experimental groups, with 1 mg/mL of Pac-525 solution as positive control group and PBS as negative control group), and then incubated at 37 °C for 16–18 h. Afterwards, 100 μL of the mixture solution was transferred to a 96-well cell culture plate and examined using a multifunction microplate reader to record the optical density value at 620 nm. The inhibition ratio (*R*) was calculated according to a formula expressed as *R* = (*A* − *B*)/*A* × 100%, where A represents the OD values of the control group (bacteria solution) and B represents the OD values of the experimental group (mixture of bacteria solution and testing solution). Generally speaking, when the inhibition rate is between 50% and 90%, it means that the sample has an antibacterial effect, and if the result is over 90%, it indicates that the sample has a bactericidal effect.

#### 2.2.9. Statistical Analysis

All the data are expressed as mean ± SD. The statistical analysis was performed with one-way analysis of variance and Student’s *t*-test. The data were considered statistically significant when *p* < 0.05. All of the data were analyzed using SPSS 13.0 software (SPSS China, Shanghai, China) for Windows, Student Version.

## 3. Results

### 3.1. Characterization of the Gln/CS Composite Membranes

**Figure 2:**
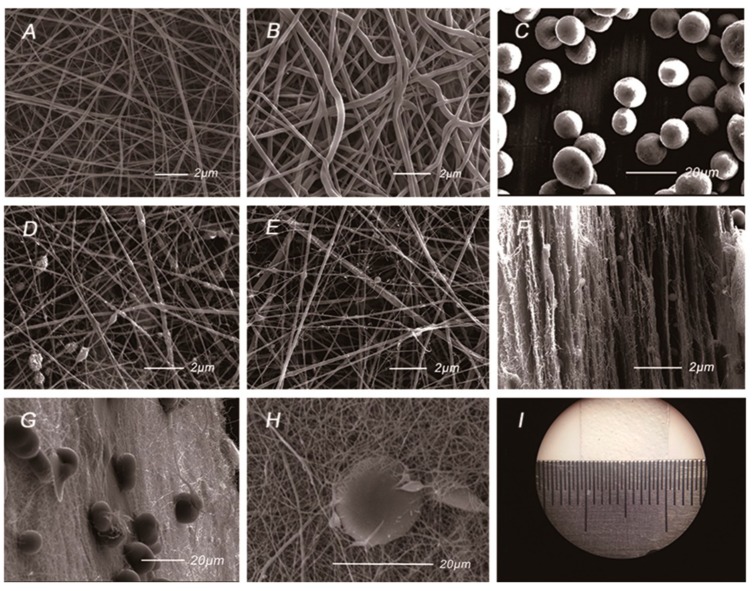
The gross and SEM morphologies of the Gln/CS composite membrane. (**A**) The barrier layer of Gln/CS nanofibers before crosslinking; (**B**) The barrier layer of Gln/CS nanofibers after crosslinking; (**C**) Electrosprayed AMP@PLGA microspheres; (**D**,**E**) The osteogenic layer of Gln/CS/nHAp by magnetic stirring (**D**) and ultrasonic dispersion (**E**); (**F**) The typical cross-sectional morphology of the membrane; (**G**,**H**) The typical morphologies of the AMP@PLGA MSs embedded within the membrane; (**I**) Gross image of the Gln/CS composite membrane.

The AMP@PLGA-MS@Gln/CS/nHAp membrane was prepared by sequential layer-by-layer electrospinning and electrospraying techniques, as sketched in Figure 1. The gross and SEM morphologies of the membrane are shown in Figure 2. Figure 2A,B shows the uniform and interwoven nanofibers of the barrier layers prepared by electrospinning of 14% *w*/*v* Gln/CS solutions in acetic acid before and after crosslinking, the diameters of which were 359 ± 174 nm and 409 ± 197 nm, respectively. The introduction of crosslinking agent significantly increased the fiber diameter (*p* < 0.05). Figure 2D–F shows the typical morphologies of the osteogenic layer containing hydroxyapatite nanoparticles. It’s noted that the nHAp was able to more uniformly disperse in the electrospun nanofibers by ultrasonic dispersion than magnetic stirring. Figure 2F shows the typical cross-sectional morphology of the composite membrane, displaying the sequential nanofibrous layers with embedded PLGA-MSs. The eletrosprayed PLGA microspheres embedded within the layers can be clearly seen in Figure 2G,H. The gross image of the GBR membrane is shown in Figure 2I, as well.

The tensile strengths of Gln/CS and Gln/CS/nHAp fibrous layers were 3.15 ± 0.57 and 4.97 ± 2.43 MPa, respectively. After crosslinking, the strength of the Gln/CS and the Gln/CS/nHAp membranes were increased to 5.11 ± 0.96 and 7.22 ± 1.49 MPa, respectively. As shown in Table 1, the introduction of nHAp increased the strength, although the difference was not statistically significant, and the use of a crosslinking agent markedly increased the strength (*p* < 0.05). The water contact angles measurement showed that WCA of Gln/CS layer was 73.17 ± 1.22°, while the WCA of Gln/CS/nHAp layer was 69.53 ± 0.31°.

### 3.2. In Vitro Biocompatibility

**Figure 3:**
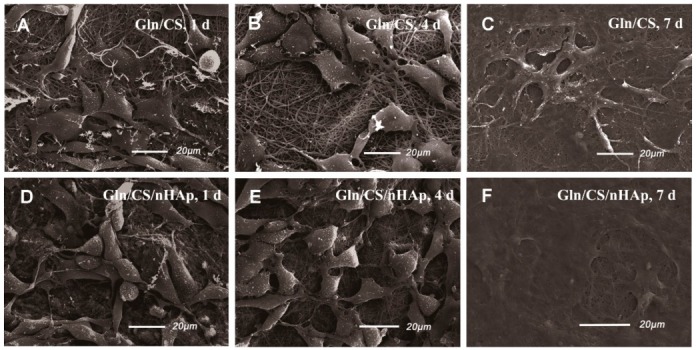
SEM micrographs of rBMSCs seeded on the barrier layer (**A**–**C**) and osteogenic layer (**D**–**F**) of the Gln/CS composite membrane after 1 d, 4 d, and 7 d of cell culture.

**Figure 4:**
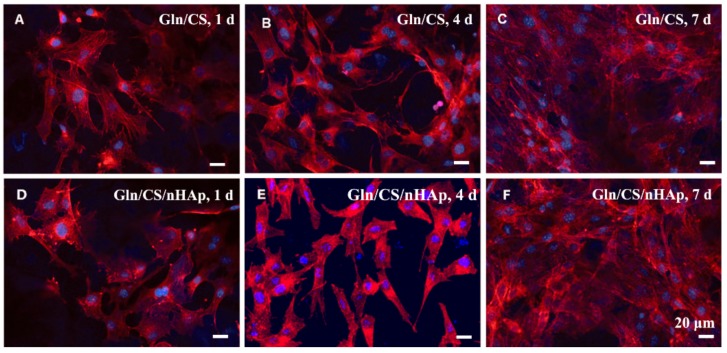
CLSM micrographs of rBMSCs seeded on the barrier layer (**A**–**C**) and osteogenic layer (**D**–**F**) of the Gln/CS composite membrane after 1 d, 4 d, and 7 d of cell culture.

**Figure 5:**
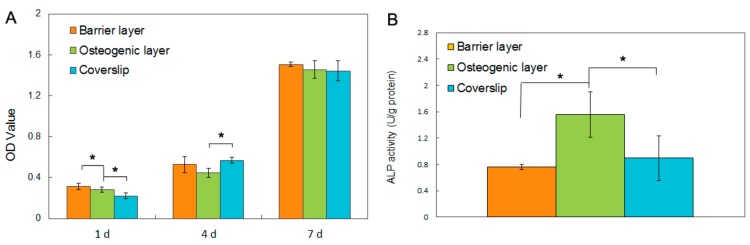
Cell behaviors of rBMSCs cultured on the Gln/CS composite membrane. (**A**) Cell proliferation on the barrier layer, osteogenic layer and coverslip control by CCK-8 assay; (**B**) Osteogenic differentiation of rBMSCs on the barrier layer, osteogenic layer and coverslip control by ALPase activity assay. * *p* < 0.05.

The biocompatibilities of the two types of fibrous layers Gln/CS and Gln/CS/nHAp were evaluated by in vitro cell culture of rBMSCs. The typical cell morphologies cultured on Gln/CS and Gln/CS/nHAp layers at 1, 4, and 7 days are shown in Figure 3 (SEM images) and Figure 4 (CLSM images). It’s revealed that rBMSCs had good attachment and spreading with typical spindle-like shapes on the barrier layer and osteogenic layer, indicating that both the Gln/CS and Gln/CS/nHAp layers had good biocompatibility. Additionally, the rBMSCs proliferated gradually and reached confluence at 7 days of cell culture. Cell proliferation was also evaluated quantitatively by CCK-8, as shown in Figure 5A. It could be clearly seen that the cells underwent very good proliferation for all the sample groups. More than that, the cell number on the barrier or osteogenic layer was significantly higher than that on coverslip control at day 1 after cell seeding, which also confirmed the good cell attachment and viability on the electrospun composite membrane. Additionally, the involvement of AMP@PLGA-MS didn’t obviously change the cell morphologies, indicating good biocompatibility of AMP-loaded PLGA microspheres (data not shown).

### 3.3. Alkaline Phosphatase (ALPase) Activity Assay

To investigate the osteogenic activity of the electrospun membrane, the rBMSCs were seeded onto the two kinds of fibrous layers with and without nHAp and cultured in the osteogenic culture medium for 14 days. The ALPase activity was evaluated, as shown in Figure 5B. It’s noted that the ALpase activity on the Gln/CS/nHAp layer was significantly higher than that on the Gln/CS layer and coverslip control group, which indicates that the involvement of nHAp was able to promote osteogenic differentiation of rBMSCs in comparison with the fibrous Gln/CS matrix without nHAp.

### 3.4. In Vitro Drug Release Profile

**Figure 6:**
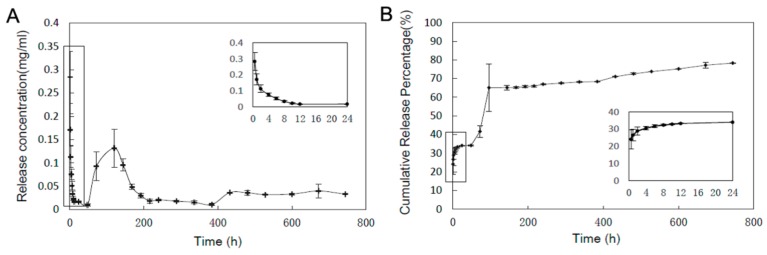
In vitro release profile of Pac-525 from the Gln/CS composite membrane. (**A**) The concentration of released Pac-525; (**B**) Cumulative release percentage of Pac-525.

The in vitro release profile of the antimicrobial peptide Pac-525 from the GBR membrane was evaluated, as depicted in Figure 6. It should be noted that there was a quick release in the first 24 h because of the peptides on the surface of the PLGA microspheres, and the cumulative release rate reached 35%. After the first burst release, the AMPs showed sustained and moderate release, then had a second burst release with a cumulative release rate of 65% at around 4 days of incubation. Considering the swelling of the PLGA MSs encapsulated in the thin membrane, it’s estimated that the AMPs in the near surface of the PLGA MSs diffused out quickly, resulting in the second burst release. Subsequently, the AMPs were released slowly, mainly accompanying the biodegradation of PLGA.

### 3.5. Antibacterial Property

**Figure 7:**
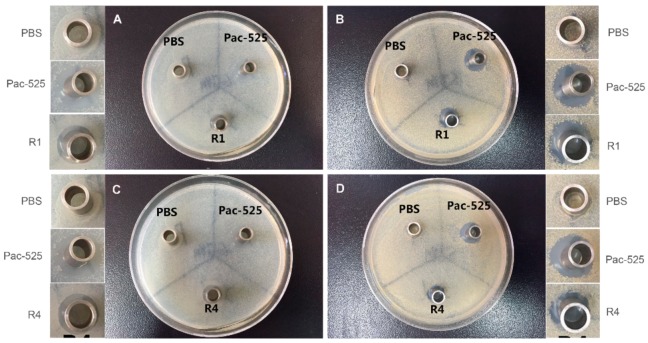
The typical morphologies of the inhibition zone induced by the elution solutions of AMP@PLGA-MS@Gln/CS/nHAp composite membrane at the first week (**A**,**B**) and the fourth week (**C**,**D**) against *E. coli* (**A**,**C**) and *S. aureus* (**C**,**D**) after 3 days of incubation on agar plate.

The antibacterial activities of the released AMPs against *S. aureus* and *E. coli* were evaluated using inhibition zone and inhibition ratio assays. As displayed in Figure 7, a typical morphology of the inhibition zones was observed surrounding the Oxford cups, in which the control solutions (PBS or prepared Pac-525 solution) or the elution solutions of AMP-loaded GBR membranes from various time intervals between 1 week and 4 weeks were loaded. The obvious inhibition zones were shown in the sample groups and the positive control group again *E. coli* (Figure 7A,C) and *S. aureus* (Figure 7B,D), while no such inhibition zones were observed in the PBS control group. The diameter of the antibacterial ring was measured, and the obtained results are as follows: the diameter of the positive control group against both *E. coli* and *S. aureus* was about 14 mm; in the sample group with 1 wk-elution solution, it was 11 and 12 mm against *E. coli* and *S. aureus*, respectively; in the sample group with 4-wk-elution solution, it was 9 and 11 mm against *E. coli* and *S. aureus*, respectively. It’s noted that the diameters of the inhibition zones decreased in the sample groups from one week to four weeks of incubation because of the decreased concentration of released Pac-525 in the elution solution.

Additionally, the inhibitory ratios were also measured, as listed in Table 2. The inhibitory ratio of the 1-wk-elution solution against *E. coli* and *S. aureus* was 94.61% and 95.08%, respectively. As for the 4-wk-elution solution, it was 68.26% and 77.36%, respectively. These results demonstrated that the GBR membrane had a bactericidal effect on *E. coli* and *S. aureus* in the first week, and still had an antibacterial effect four weeks later.

## 4. Discussion

Insufficient bone mass and infection are two main reasons for the dental implant failure. The aim of the current study is to fabricate an electrospun anti-infective GBR membrane loaded with nHAp and AMPs that would be favorable for bone regeneration, in addition to decreasing bacterial colonization and preventing infection over a long period of time. For this purpose, the electrosprayed AMP-loaded microspheres were embedded in an electrospun Gln/CS nanofiber membranes with or without nHAp by alternate layer-by-layer electrospraying.

In the present study, the GBR membrane was designed to possess a bilayer structure consisting of barrier and osteogenic layers, similar to the well-known Bio-Gide collagen membrane (Geistlich Pharma AG, Switzerland) [22,23,24]. Although the Bio-Gide collagen membrane has shown good effects in many applications, including bone augmentation, alveolar ridge reconstruction, and guided bone regeneration, it lacks sufficient antibacterial and osteogenic activities. Here, the barrier layer of our BGR membrane was composed of interwoven nanofiberous Gln/Cs that could be placed next to the soft tissues to prevent the invasion of soft tissues, thus maintaining the defective space as well as protecting the newly formed bone tissue. At the same time, the nHAp was added, serving as a mineral reservoir of calcium and phosphate ions to construct an osteogenic layer that is placed toward the bone defect, stabilizing the blood clot and providing a proper support for the adhesion of osteoblasts and the deposition of new bone as well. Furthermore, in order to endow the membrane with antibacterial activities, a new AMP, Pac-525 was introduced into the membrane. To prevent the peptide from being damaged during the preparation process, a PLGA microsphere drug delivery system was used to protect the antimicrobial peptide and maintain its long-term effectiveness because of its good biocompatibility and adjustable biodegradability [25,26,27,28,29,30]. Additionally, the positively charged CS was used here because of its broad range of antimicrobial behavior [31]. To sum up, the design and schematic diagram of the AMP-loaded bilayer Gln/CS GBR membrane is shown in Figure 1.

In order to overcome the problem of aggregation of nHAp in fibers, the method of ultrasonic dispersion was used to evenly disperse the particles. The SEM images showed the uniform Gln/CS nanofiber with the nHAp particles homogeneously dispersed and arranged along it. The incorporation of nHAp slightly improved the hydrophilicity of the Gln/CS membrane because of hydrophilic ions on the hydroxyapatite’s surface, which is appropriate for cell attachment and cytoskeletal spreading. At the same time, the nHAp dispersed in the nanofibrous Gln/CS membrane significantly promoted osteogenic differentiation of rBMSCs. Nano-hydroxyapatite has proper biocompatibility and osteoconductivity, and has been widely used in bone regeneration by improving the function of osteoblasts [32]. The ALP activity tests showed that the cells cultured on the surface of Gln/CS/nHAp membranes had high expression of ALP activity, indicating that Gln/CS/nHAp membranes could provide a suitable environment for cell growth and promote osteogenic differentiation of MSCs.

On the other hand, the initial burst release and subsequent sustained release of Pac-525 from the AMP-loaded GBR membrane contributed to the membrane a certain bactericidal activity in a short time and antibacterial activity over a long period of time against two kinds of bacteria, *S. aureus* and *E. coli. S. aureus* represents the Gram-positive bacteria, and *E. coli* is a representative of the Gram-negative bacteria. They can represent the condition of most bacteria to a certain extent. The bactericidal activity of the membrane has great clinical significance, killing the pathogen in order to prevent early postoperative infection. Moreover, the long-term antibacterial activity can inhibit the proliferation of bacteria and reduce the incidence of infection.

Furthermore, considering the clinical application of GBR membranes, the resistance to tear under tensile stresses was also one of the important properties of the membranes. The tensile strength of the electrospun Gln/CS and Gln/CS/nHAp fibrous matrices were 3.15 ± 0.57 and 4.97 ± 2.43 MPa, respectively. In order to improve the mechanical property and stability in aqueous environment, chemical crosslinking was conducted. After crosslinking, the tensile strength of the Gln/CS and the Gln/CS/nHAp fibrous matrices were increased to 5.11 ± 0.96 and 7.22 ± 1.49 MPa, respectively. Both the introduction of nHAp and chemical crosslinking increased the tensile strength of the membrane. The mechanical properties of the electrospun GBR membrane met the general requirements for the GBR membranes as previously reported in the literature [33]. The chitosan macromolecule with a rigid backbone and the NH^2+^ and OH^-^ groups was entangled with gelatin molecules and had strong intermolecular interactions through the hydrogen bonds and crosslinked covalent bonds, which probably contributed to the good mechanical properties of the composite Gln/CS membrane.

Although the AMP@PLGA-MS@Gln/CS/nHAp bilayer membrane exhibited excellent osteogenic and antibacterial properties in vitro, additional studies should be carried out to further evaluate the importance of the bilayer membrane on leading bone regeneration and inhibit infection in vivo.

## 5. Conclusions

A gelatin and chitosan composite nanofibrous membrane containing nHAp and AMP-loaded PLGA microspheres was prepared by sequential layer-by-layer electrospinning and electrospraying techniques, and then evaluated by examining its morphology, mechanical properties, biocompatibility, osteogenic activity, and antibacterial properties. The SEM images showed the uniform electrospun Gln/CS/nHAp nanofibers embedded with PLGA-MS. Both the involvement of nHAp and chemical crosslinking could improve the fiber diameter and tensile strength. The Gln/CS composite membrane had an ideal biocompatibility with good cell adhesion, spreading, and proliferation. In addition, the Gln/CS membrane containing nHAp could promote osteogenic differentiation of rBMSCs. According to the in vitro drug release assay and antibacterial experiments, the composite GBR membrane containing AMP-loaded PLGA microspheres had a long-term sustained release of Pac-525, which had bactericidal activity over one week and antibacterial activity over a long period of time against two kinds of bacteria, *S. aureus* and *E. coli*.

## Figures and Tables

**Table 1:** WCA and mechanical property of the barrier and osteogenic layers.

Test	Type	Barrier Layer	Osteogenic Layer
WCA (°)	-	73.17 ± 1.22	69.53 ± 0.31
Tensile Strength (Mpa)	crosslinked	5.11 ± 0.96	7.22 ± 1.49
	un-crosslinked	3.15 ± 0.57*	4.97 ± 2.43*

* *p* < 0.05, vs. the same type of membrane layer after crosslinking.

**Table 2:** The inhibition ratios and diameter of inhibition ring of the elution solutions extracted with different incubation times against *E. coli* and *S. aureus*.

Bacterial Species	Inhibition Ratio		Diameter of Inhibition Ring	
	1st wk	4th wk	1st wk	4th wk
*E.coli*	94.61%	68.26%	11 cm	9 cm
*S. aureus*	95.08%	77.36%	12 cm	11 cm
